# Comparison of cellular and humoral immune responses induced by primary, consensus or mosaic HIV-1 Env DNA vaccines

**DOI:** 10.1186/1742-4690-9-S2-P333

**Published:** 2012-09-13

**Authors:** J Yan, P Pankhong, AS Khan, NY Sardesai, DB Weiner

**Affiliations:** 1 Inovio Pharmaceuticals, Blue Bell, PA, USA; 2 University of Pennsylvania, Philadelphia, PA, USA

## Background

The genomic variability of HIV viruses has impeded the development of globally relevant HIV vaccines with ability to induce strong and broad cellular and humoral immune responses. Many strategies, such as codon/RNA optimization, addition of highly efficient leader sequences, use of consensus or mosaic antigens, and electroporation (EP) have all been applied to enhance the breadth and magnitude of immune responses induced by DNA vaccines.

## Methods

Highly optimized clade B and C consensus envelope DNA vaccines (pEY2E1-B and pEY3E1-C) were developed. Their immunogenicity was compared to the immune responses induced by an optimized mosaic gp160 envelope vaccine (pMosEnv) or optimized primary clade B and C envelope vaccines (pPK61-14 and p96ZM651gp140-CD5). The immunogenicity of these constructs was studied in different murine models. All vaccines were delivered using electroporation. Antibody responses were determined by ELISA and cellular responses by IFN-γ ELISPOT.

## Results

Antibody analysis supported that the consensus immunogens induced stronger clade-specific antibody response than the primary immunogens, while the mosaic antigen was a poor inducer of antibody responses. Compared to the primary vaccine constructs, both consensus and mosaic constructs were more potent at driving diverse cellular immune responses. The strongest cross-reactive immune responses against various consensus peptides libraries were induced by pEY2E1-B and pEY3E-C. The consensus immunogens were up to three times more potent at driving subtype-specific responses that recognized the different cross-clade immunogens. pMosEnv exhibited robust cellular responses when a PTE peptide set was used.

## Conclusion

We conclude that the highly optimized consensus immunogens may fold into a relatively native conformation and exhibit improved focus on antibody and T cell conserved regions, while the mosaic immunogen provides important T cell responses but does not preserve the native structure of envelope to allow for similar antibody responses in their current forms. Further exploration of these immunogens will be interesting.

